# Synthetic Polyploidy in Grafted Crops

**DOI:** 10.3389/fpls.2020.540894

**Published:** 2020-11-05

**Authors:** Marta Ruiz, Julie Oustric, Jérémie Santini, Raphaël Morillon

**Affiliations:** ^1^Centro de Protección Vegetal y Biotecnología, Instituto Valenciano de Investigaciones Agrarias, Moncada, Spain; ^2^Department of Botany and Plant Sciences, University of California, Riverside, Riverside, CA, United States; ^3^Laboratoire Biochimie et Biologie Moléculaire du Végétal, CNRS, UMR 6134 SPE, Université de Corse, Corte, France; ^4^CIRAD, UMR AGAP, Equipe SEAPAG, F-97170 Petit-Bourg, Guadeloupe, France - AGAP, Univ Montpellier, CIRAD, INRAE, Institut Agro, Montpellier, France

**Keywords:** grafting, polyploid, rootstock, scion, stress tolerance

## Abstract

Synthetic polyploids have been extensively studied for breeding in the last decade. However, the use of such genotypes at the agronomical level is still limited. Polyploidization is known to modify certain plant phenotypes, while leaving most of the fundamental characteristics apparently untouched. For this reason, polyploid breeding can be very useful for improving specific traits of crop varieties, such as quality, yield, or environmental adaptation. Nevertheless, the mechanisms that underlie polyploidy-induced novelty remain poorly understood. Ploidy-induced phenotypes might also include some undesired effects that need to be considered. In the case of grafted or composite crops, benefits can be provided both by the rootstock’s adaptation to the soil conditions and by the scion’s excellent yield and quality. Thus, grafted crops provide an extraordinary opportunity to exploit artificial polyploidy, as the effects can be independently applied and explored at the root and/or scion level, increasing the chances of finding successful combinations. The use of synthetic tetraploid (4x) rootstocks may enhance adaptation to biotic and abiotic stresses in perennial crops such as apple or citrus. However, their use in commercial production is still very limited. Here, we will review the current and prospective use of artificial polyploidy for rootstock and scion improvement and the implications of their combination. The aim is to provide insight into the methods used to generate and select artificial polyploids and their limitations, the effects of polyploidy on crop phenotype (anatomy, function, quality, yield, and adaptation to stresses) and their potential agronomic relevance as scions or rootstocks in the context of climate change.

## Highlights

- Grafting improves agronomic traits by combining well adapted rootstocks and improved scions.
- Polyploidy induces large changes in anatomical traits in the rootstock as well as in the scion.
- Polyploidy of the rootstocks and scions may contribute to stress adaptation.
- Phenotypic traits in polyploids are often associated with large physiological, biochemical, transcriptomic, and gene expression changes.

## Introduction

Polyploidy is one of the main factors driving evolution in higher plants (Grant, 1981; Soltis and Soltis, 1995, 2009; Wendel and Doyle, 2005; Cui et al., 2006; Chen, 2007, 2010; Husband et al., 2008; Hollister et al., 2012), conferring genotypic plasticity by increasing the number of copies of the genome (autopolyploidy) or adding different genomes (allopolyploidy), thus increasing their potential for adaptation (Leitch and Leitch, 2008) and promoting their selection (Feldman and Levy, 2012). It has been proposed that polyploidy favors adaptive evolution to changing environmental conditions (Ramsey, 2011) through differential expression of duplicate genes (Dong and Adams, 2011; Tan et al., 2015).

Better adaptive plasticity was found in natural polyploid plants allowing successful domestication events for many species under natural growing conditions (Salman-Minkov et al., 2016) such as autotetraploid wheatgrass [*Agropyron desertorum* (Fisch. ex Link) Schult.], potato (*Solanum* spp.), and allopolyploid wheat (*Triticum* spp.; DeWet, 1980; Asay et al., 1986; Liu et al., 2001). However, when considering human-made polyploidy, only a few events have achieved commercial success, like autotriploid watermelon (*Citrullus vulgaris* Schard.), autotriploid sugar beet (*Beta vulgaris* L.), autotetraploid kiwi (*Actinidia chinensis* Planch), auto-allopolyploid apple (*Malus* spp. Mill), banana (*Musa* spp.), and grape (*Vitis* spp.; Yamane and Kurihara, 1980; Crow, 1994; Janick et al., 1996; Motosugi et al., 2002; Wu et al., 2012).

**Figure 1:**
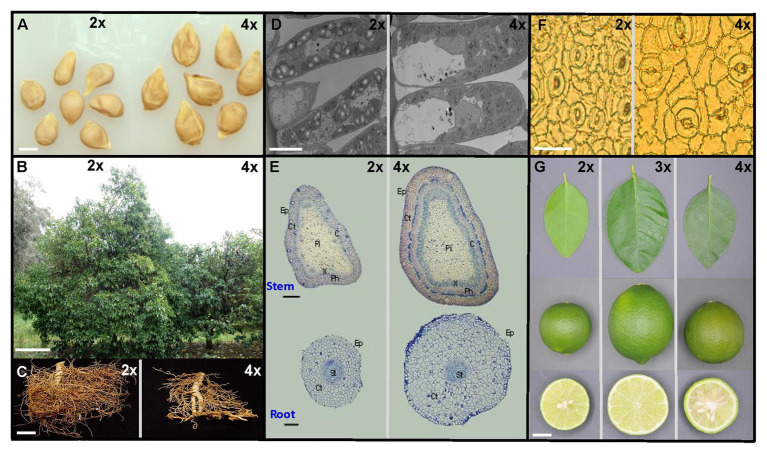
Illustration of the phenotypic differentiation between diploid (2x) and polyploid (triploid, 3x and tetraploid, 4x) citrus at the plant, organ and cellular level. **(A)** Diploid and 4x seeds of Citrumelo (bar = 0.5 cm). **(B)** Diploid and 4x Citrumelo trees planted at the same age at the INRAe – Cirad germplasm of San Giuliano, France (bar = 0.5 m). **(C)** Diploid and 4x Carrizo citrange fibrous roots (bar = 1 cm). **(D)** Scanning electron microscopy pictures of the leaf palisade parenchyma of 2x and 4x Volkamer lemon (bar = 10 μm). **(E)** Light micrographs of cross-sections of internodes and roots of Rangpur lime plants (bar = 25 μm) from Allario et al. (2011). **(F)** Light micrographs of leaf epidermis showing stomata from 2x and 4x Citrumelo (bar = 10 μm). **(G)** Leaf and fruit of Mexican lime (2x), Tahiti lime (3x), and Giant Mexican lime (Autotetraploid; bar = 0.5 cm).

In agriculture, the genomic modifications that take place during polyploidization confer many interesting advantages over the diploid (2x). The most important for crop production are dwarfing effect on trees, increase in organ biomass (leaves, fruit, seeds, roots, etc.), alteration of flowering time, intensification of color (leaves and fruit), such as illustrated in Figure 1 and detailed in Table 1, increased primary and secondary metabolite content and enhanced tolerance or resistance to abiotic and biotic stresses (see Polyploidy Improves Stress Tolerance section). In addition, triploidization can limit gametic fertility due to unbalanced meiosis. Associated with parthenocarpy, this reduced fertility allows the production of seedless fruit, which is a desirable trait for consumers. Polyploidization can also restore fertility in newly created hybrids (Sattler et al., 2016). Thus, the success of polyploidization as a tool is highly dependent on the crop species and is shaped by commercial interests such as biomass production, ornamental crops, or the pharmaceutical industry.

**Table 1:** Selection of polyploid crops that have been reported for their agronomic interest.

Crop	Ploidy	Agronomic traits	References
Apple	3x, 4x	Increased fruit size and dwarfism^1^^,^^2^	Janick et al., 1996; Sedov, 2014; Ma et al., 2016
	4x	Drought tolerance	De Baerdemaeker et al., 2018
Birch	4x	Increased biomass and dwarfism	Mu et al., 2012
Black locust	4x	Salinity tolerance^1^	Wang et al., 2013a; Meng et al., 2016; Luo et al., 2017
Blackcurrant	4x	Increased fruit size	Podwyszyńska and Pluta, 2019
Citrus	4x	Drought, salinity, and boron excess tolerance^2^^,^^3^; cold, nutrient deprivation and chromium toxicity tolerance	Allario et al., 2013; Podda et al., 2013; Ruiz et al., 2016a; Balal et al., 2017; Oustric et al., 2017, 2018, 2019a; Khalid et al., 2020
	4x	Dwarfism^1^^,^^2^^,^^3^	Hussain et al., 2012; Grosser et al., 2015; Ruiz et al., 2016a
	3x	Increased fruit quality, size and seedlessness^1^^,^^2^	Ollitrault and Navarro, 2012; Tan et al., 2019; Sdiri et al., 2020
Cherimoya	3x	Increased fruit size	Martin et al., 2019
Fig tree	3x	Increased fruit size	Falistocco, 2016
Forest mangrove	3x, 4x	Increased biomass yield and quality	Harbard et al., 2012; Griffin et al., 2015
Grape	3x, 4x	Increased fruit size and seedlessness^1^^,^^2^	Yamada and Sato, 2016
Kiwi	4x	Increased fruit size^1^	Wu et al., 2012; Zhong et al., 2012
Loquat	3x, 4x	Increased fruit size	Jiang et al., 2016
Mango	4x	Increased fruit size; enhanced photoprotection	Galan-Sauco et al., 2001; García-García et al., 2020
Mulberry	3x, 4x	Increased fruit size and leaf biomass yield	Dai et al., 2015; Shafiei, 2018
Olive	4x	Increased fruit size	Rugini et al., 2016
Pawlonia	4x	Increased biomass yield and quality; salinity and drought tolerance	Tang et al., 2010; Zhao et al., 2017
Persimmon	6x	Increased fruit size^1^	Yesiloglu et al., 2018
Pomegranate	4x	Increased fruit size	Shao et al., 2003
Sweet cherry	3x	Dwarfism^1^^,^^2^	Prassinos et al., 2009
Watermelon	4x	Higher vigor and resistance to the RKN^1^^,^^2^^,^^3^	Levi et al., 2014
	3x, 4x	Increased fruit size and quality	Jaskani et al., 2004; Zhang et al., 2019
Willow	3x, 4x	Increased biomass yield and quality; dwarfism	Serapiglia et al., 2015; Dudits et al., 2016

1 Commercial success.

2 Tested grafted.

3 Tetraploid tested as a rootstock.

Grafting has been used for centuries to propagate fruit trees. In vegetable crops, the technique has been used mainly since the beginning of the 20th century. Grafting of a scion or crop variety onto a rootstock is effective at providing faster and more regular growth in commercial orchards, facilitating earlier development by shortening juvenility, improving yield and fruit quality, modulating the harvest season (Koepke and Dhingra, 2013), and providing abiotic stress tolerance and resistance to specific biotic stress (Mudge et al., 2009). In some perennial plants, grafting helps to maintain uniformity through clonal propagation of both scion and rootstock and in some cases, it limits the impact of the juvenile phase. Thus, combining grafting impacts with the use of a polyploid rootstock and scion might bring great advantages in cultivated crop.

Climate change is already challenging agriculture (Intergovernmental Panel on Climate Change, IPCC – Fifth Assessment Report 2014; IPCC, 2014; Thornton et al., 2018). It will result in higher temperatures, drought, and increased soil salinity (Korres et al., 2016). As a major force for plant evolution (Chen, 2010), polyploidy promotes better adaptation traits in crops, since polyploid plants are thought to have been selected during evolution because of their phenotypic and genomic plasticity (Leitch and Leitch, 2008). Much larger proportions of polyploid plants have been found in the Arctic (Brochmann et al., 2004) and at mountainous elevations (Schinkel et al., 2016), suggesting that these genotypes are better adapted to severe cold climatic constraints. However, these populations such as paleopolyploids may have experienced large genome changes leading to a loss of their polyploid status.

The information presented in this review regarding the adaptation traits in synthetic polyploids is consistent with that observed in natural polyploid plant populations and underlines the importance of pursuing investigations in synthetic polyploidy for grafted crops. The use of polyploid rootstocks and scions brings up numerous questions on the genetic, transcriptomic, physiological, and agronomic level. Thus, we addressed (i) the methods to create artificial polyploids; (ii) the reproductive biology implications that using polyploid rootstocks and scions may have on crops; (iii) the importance of grafting in agriculture and the implications of using polyploid crops; (iv) the phenotypic variation induced by polyploidy and its effect biomass production and fruit; (v) the implications that polyploidy has in the regulation of genome expression with a focus on fruit quality and stress tolerance; and (vi) the role of polyploidy for enhancing stress tolerance.

## Methods to Generate Polyploids and Reproductive Biology Implications

Associated to breeding programs, different methods have been developed to generate polyploids that require having a good knowledge of the reproductive biology of the investigated species. Several advantages and disadvantages have been associated with each one of the approaches.

The oldest method used to generate polyploids is sexual polyploidization. It is based on controlled pollination that involves at least one of the parents providing non-reduced gametes (usually 2n) as a consequence of meiotic aberrations. First-division restitution (FDR) and second division restitution (SDR) are the predominant mechanisms of 2n gamete formation in plants (De Storme and Geelen, 2013). These occur because the first or second meiotic divisions fail, respectively, leading to the formation of restituted nuclei with a somatic chromosome number (Mendiburu and Peloquin, 1976; Park et al., 2007). As a result, FDR and SDR 2n gamete formation mechanisms have different genetic implications that are worth considering for polyploid breeding because it has a direct influence on the genetic constitution of the progeny. FDR 2n gametes contain non-sister chromatids, which in the absence of crossover, maintain the parental heterozygosity. When crossover occurs, the parental heterozygosity restitution (PHR) rates vary from 100% for loci close to the centromere to 60–70% for loci far from the centromere, depending on the level of chromosome interference (Cuenca et al., 2011). For SDR, the 2n gametes contain two sister chromatids, which reduces the parental heterozygosity level (Bastiaanssen et al., 1998; Cuenca et al., 2011; De Storme and Geelen, 2013). In this case, when crossover occurs, the PHR rate varies from 0% for loci close to the centromere to 60–75% for loci far from the centromere, depending on the level of chromosome interference (Cuenca et al., 2011). In the genus *Annona*, which includes edible fruits like cherimoya (*A. cherimola* Mill.) and sugar apple (*A. squamosa* L.), the production of non-reduced gametes has been identified after unusual polyploid progenies were observed (Martin et al., 2019). Also, analyses based on molecular markers can be used to estimate the PHR rates for 2n gametes in polyploid progenies and, therefore, to identify the mechanisms underlying unreduced gamete formation (Cuenca et al., 2011, 2015). The main limitation of sexual polyploidization is that non-reduced gametes are usually produced by plants at a very low rate, when abnormal meiosis is induced by genetic or environmental factors, such as temperature, herbivory, wounding, water deficit, or nutrient shortage (Ramsey and Schemske, 1998). However, unlike somatic methods, sexual polyploidization is effective at preventing somaclonal variation on the progenies.

Another method to generate polyploid plants is interploid sexual hybridization, which involves crossing plants that have different ploidy levels. This is a very common approach to recover triploid (3x) plants by 2x × 4x, 4x × 2x, or 2x × 3x crosses. However, it has the disadvantage of frequent endosperm development failure and hence seed abortion (Birchler, 2014). For this reason, embryo rescue is an indispensable technique for breeding programs based on interploid crosses (Wang et al., 2016); it has been applied extensively in many fruit crops, such as apple (Dantas et al., 2006), citrus (Aleza et al., 2012), or grape (Sun et al., 2011). Li et al. (2015) provided an overview of the factors that may affect its efficiency.

Polyploidization can also be induced by somatic doubling, which involves chromosome duplication in non-germ cells to generate autopolyploidy or allopolyploidy, according to the phylogenomic structure of the initial 2x accession. In this case, duplication arises because of a mitotic failure and can be chemically induced by antimitotic agents, such as colchicine, trifluralin, and oryzalin. However, in crops that have nucellar embryony, such as several citrus and mango (*Mangifera* spp.) species, the spontaneous somatic duplication events occurring in the nucella may result in the natural development of tetraploid (4x) embryos that can be selected from seedlings (Galan-Sauco et al., 2001; Aleza et al., 2011). When somatic duplication is induced chemically, success depends largely on the development of an effective protocol that sets the proper explant type, antimitotic agent dose, exposure time, and *in vitro* regeneration conditions. Ploidy chimeras are often a secondary effect induced by chemical treatments. Therefore, this method requires further verification of the ploidy of different organs on each regenerant explant that usually involves morphological examination, karyotyping, or flow cytometry analytical approaches. Somatic doubling has been extensively used for polyploid crop breeding, as reviewed by Sattler et al. (2016).

Lastly, somatic hybridization by protoplast fusion, which was initially developed to overcome crossing barriers between species, allows the whole genomes of two different parents to be combined in a single cell that can be regenerated into a hybrid plant that is usually polyploid. This method allows polyploids that have not been through meiotic recombination events to be generated. Thus, progenies combine the whole genomes of the two parents and, potentially, all the dominant parental traits, irrespective of their heterozygosity and surpassing limitations imposed by reproductive biology. Meanwhile, the probability of combining all the desired traits in a sexual recombinant hybrid is much lower and slower on crops with long juvenile stage (Dambier et al., 2011; Ollitrault and Navarro, 2012). Difficulties in protoplast isolation, culture, and plant regeneration hinder the use of this approach in many crops. However, it is an integrated component of several citrus breeding programs over the world, both for scion and rootstock breeding (Grosser et al., 2000; Dambier et al., 2011; Grosser and Gmitter, 2011).

Once obtained, artificial polyploids can be classified either as breeding material or considered as potential new varieties. Hence, their potential benefits and disadvantages need to be examined relative to their prospective use, in order to maximize the potential of each new developed genotype. Triploids are mainly valuable for having bigger seedless fruit, while their limited fertility usually acts as a dead end for further improvement. The way to add required traits such as disease resistance *a posteriori* into a 3x variety is to resynthesize it using improved parents (de Carvalho-Santos et al., 2019). More importantly, 3x crops need to be able to activate a parthenocarpic fruit set, which can be stimulated by pollination, induced by hormonal treatments or through epigenetic manipulation (Joldersma and Liu, 2018). Fortunately, natural parthenocarpy is very common among cultivated species, especially on trees, plants of hybrid origin, and polyploids (Picarella and Mazzucato, 2019).

Tetraploids (4x) are widely used as progenitors and are often included in germplasm collections to assist in breeding programs. As a genetic resource, 4x can be used to generate 3x varieties through interploidy crosses or to serve as a bridge for genetic transfer between two species when direct crossing is not possible (Liu et al., 2017). According to Sattler et al. (2016), genome duplication can also be induced to restore the fertility of sterile hybrids as can restore meiosis and can buffer the effect of deleterious alleles. In addition to this, 4x genotypes are a valuable resource when used as crops that are cultivated for their vegetative organs because biomass generally increases with genome duplication. Also, 4x can be very useful as rootstocks because genome duplication can enhance some desired traits like stress resistance or canopy size control. However, delayed flowering and poor fruit quality are recurrent phenotypes observed in autotetraploid and allotetraploid species that hamper their use for fruit production.

Vegetative propagation is usually a requirement for both 3x and 4x varieties, as 4x usually have lower rates of seed production, thus clonal multiplication is needed to maintain the genotype. The enhancement of asexual reproduction is a common consequence of polyploidy in many species (Comai, 2005) and is required for crops that are cultivated grafted. Thus, suitability for clonal propagation might be facilitated at higher ploidies. Another essential point is that polyploidization can be a way to overcome incompatibility and self-incompatibility (Entani et al., 1999), which are barriers for breeding and fruit set, respectively. However, the molecular basis for this response is still unclear.

## Phenotypic Variation Induced by Polyploidy

Morphological changes after genome duplication can be explained as a series of downstream effects triggered by the increased cell size and shape, which is directly influenced by bulk DNA amount irrespective of genic content. At the cellular level, novel traits can be as simple as a change in surface-to-volume ratio, cell size, nuclear volume, or cell cycle duration (Doyle and Coate, 2019), whereas gene expression of heterozygous loci could also be affected by dose (Finigan et al., 2012). Nevertheless, whether these cellular changes are mechanistically connected to variations affecting the whole organism’s phenotype and function remains unknown. Additionally, it is sometimes difficult to distinguish between ploidy driven changes and those induced by other factors affecting artificial polyploids. To obtain, either autopolyploids or allopolyploids, it is necessary to go through processes, like chemical induction for genome duplication, protoplast fusion, *in vitro* regeneration, or the occurrence of genomic shock (Song et al., 1995) that contribute to doubt about ploidy effects and genomic identity between them and its 2x counterparts (Comai, 2005; Münzbergová, 2017).

In general terms, bigger polyploid cells can differ from 2x in their organelle number, size, and distribution, can have a higher water content or can have altered metabolic and development rates. All these modifications may have functional consequences on the plant architecture organ size and composition, physiology of essential processes, like gas exchange, photosynthesis, and water relations, probably driven by alterations of organelles such as chloroplasts and vacuoles (Doyle and Coate, 2019).

### Cell and Organ Size Modifications

A wide range of phenotypic changes at the organ level have been reported in plants due to polyploidy (Beest et al., 2012). Thicker and greener leaves, higher leaf water content, thicker and smaller roots as well as a dwarf phenotype are some of the most recurrent novel traits (Cameron and Frost, 1968; Romero-Aranda et al., 1997; Motosugi et al., 2002; Padoan et al., 2013; Ruiz et al., 2016a,b,c; Wang et al., 2016). Figure 1 illustrates some phenotypic traits induced by polyploidy in citrus and Table 1 summarizes some traits mentioned in the literature in different polyploid species. Polyploidy may also alter cell wall composition. However, this does not always change the size of the whole plant (Corneillie et al., 2019). Stomatal and epidermal cell frequency per unit leaf is usually decreased with increased ploidy level, while cell area is increased (Jellings and Leech, 1984; Beck et al., 2003; Mouhaya et al., 2010; Zhang X. Y. et al., 2010; Allario et al., 2011; Oustric et al., 2019b). Larger flowers and seeds are usually observed in polyploids such as citrus (Yahmed et al., 2016). Phenotypic changes in polyploids such as specific features of the roots or leaves may be at the origin of better tolerance traits. However, the basis for DNA amount-driven functional differentiation and the potential effects of genomic shock are poorly described, forcing those who use polyploid breeding for abiotic stress tolerance to rely on empirical methods (see Polyploidy Improves Stress Tolerance section).

### Quantitative and Qualitative Modifications on Biomass

Regarding biomass production, there is no consensus about ploidy effects on plant growth, since some species may present similar (Niu et al., 2016) or lower (Hennig et al., 2015; Denaeghel et al., 2018) organ size or biomass associated to polyploidy. Independently of plant height, polyploid plants often develop higher biomass that results in thicker tissues and organs and alter plant architecture when compared to the 2x. Several examples can be found in Table 1. There is an open discussion on whether genome duplication causes divergent changes between primary and secondary growth of woody species. While these two parameters are generally correlated in 2x species, the regulatory mechanisms coordinating plant organ growth differ between many 2x and autotetraploid trees, where dwarfism but higher biomass has been found on autotetraploid versions when compared to their corresponding 2x (Table 1). For this reason, some authors suggest genome duplication as a useful breeding tool to tailor crops for biomass production, as it has been described on the model plant Arabidopsis (*Arabidopsis thaliana* L. Heynh; Corneillie et al., 2019). In this species, polyploid biomass has an altered composition that is easier to saccharify. This property, which has largely remained unexplored for feed and food production, might pave the way for improving the efficiency and sustainability of biomass production. As an example, 3x and 4x Willow (*Salix* spp.) were found to have lower lignin content compared to the 2x parental lines (Serapiglia et al., 2015). Due to the increasing interest in using plants as a source of energy and chemical building blocks, researchers may find it useful to explore polyploidy for biomass quality improvement. Nevertheless, the mechanisms that lead genome duplication to influence plant growth, development, yield, and quality remain unanswered.

### Fruit Quality

Triploidy induces the production of seedless fruit and favors vigor (Wang et al., 2016). While triploid breeding for seedlessness in some ligneous plants such as grapevine or citrus has led to the development of outstanding varieties, some triploid apple or citrus lime have seedy fruits with more limited interest. In grape and kiwi, polyploidy increased fruit size (Shengjian et al., 2005; Wu et al., 2012).

Polyploidization can be used as a breeding tool to modify crop quality. The main effects reported are absence of seeds and modifications in fruit size, shape, and organoleptic quality. Bigger fruit size is very important commercially and is often boosted by cultural practices or chemical treatments. Polyploidization offers a natural and environmentally friendly alternative for increasing fruit size. This is a well-known effect, although the mechanisms behind it continue to be debated today. Final fruit size is determined by coordinated progression of cell production and cell expansion and is correlated with cell size, which is bigger in polyploids (Doyle and Coate, 2019). Alternatively, altered transport or signaling of auxins and cytokinins may reduce apical dominance, thereby facilitating fruit proliferation (Malladi and Hirst, 2010). Independent of the mechanism, bigger fruit size is a recurrent effect, at least for lower polyploidy levels (3x, 4x, or 6x). Recently reported examples of ploidy-driven increase in fruit size on species that are cultivated grafted are summarized in Table 1. Although, several of these recently induced polyploid forms have not yet attained the required market qualities, they comprise valuable germplasm sources for application in prospective breeding experiments.

Triploid plants are usually both male and female sterile and seeds are not viable or not present. Seedlessness is a highly desirable characteristic for consumers (Ollitrault and Navarro, 2012). Besides, triploidy increases fruit profitability in 2x species with big seeds, because seedlessness provides extra capacity for flesh (Jiang et al., 2016). Tetraploid apple varieties have no commercial value because of their low-quality fruit and low resistance to cold, being mostly used to develop the 3x cultivars (Sedov et al., 2014). The situation is similar for grapes (Yamada and Sato, 2016) and citrus (Ollitrault and Navarro, 2012). Yet, 4x germplasm is a necessary resource to obtain and maintain 3x varieties by interploid crosses. To produce 3x plants, crops like citrus or fig that are vegetatively propagated have an advantage over the ones propagated by seed, such as watermelon, that needs *de novo* interploid crosses to produce each generation.

Polyploid fruits usually have similar organoleptic and nutritional quality than their 2x counterparts. However, some studies report sporadic differences that can be relevant for commercial purposes. For example, 2x “Kinnow” mandarin has 9.5% higher total soluble solids content and 46.1% higher juice content than 4x (Jaskani et al., 2002). Similarly, 4x “Ponkan” mandarin has altered accumulation of primary and secondary metabolites in the fruit. Total acid and ascorbic acid, which are the main components of fruit flavor, are higher in the 4x fruit (Tan et al., 2019). Downregulation of gene expression involved in its transport and uptake in mitochondria like NAD-IDH, GABA-T, and GABP has been observed. Meanwhile, sucrose, fructose, and glucose content are very similar between 2x and 4x fruit, and ripening season does not differ between them, while secondary metabolites such as flavonoids and carotenoids are decreased (Tan et al., 2019). Similar results have been observed in the fruit and seeds of natural allohexaploid pitaya (*Hylocereus* spp.) and natural autotetraploid pear fruit (*Pyrus communis* L.) depending on age and growing conditions (Cohen et al., 2013; Tsukaya et al., 2015). Tetraploid kiwi has reduced flesh firmness and flesh color intensity compared with parental 2x plants (Wu et al., 2013). However, the quality of extra virgin olive (*Olea europaea* L.) oil is similar when extracted from 2x or 4x olives (Rugini et al., 2016). Not surprisingly, 3x fruits have been proven to have excellent organoleptic and nutritional quality, like is the case of mandarins (Sdiri et al., 2020). Thus, 3x is the most common ploidy in polyploid fruit breeding since it combines bigger fruit size, higher yield, and organoleptic quality with seedlessness. Additionally, some variations in specific compounds have been associated with genome duplication. This is the case for 4x watermelon, which has higher β-carotene, lycopene, fructose, and glucose content than 2x fruit (Jaskani et al., 2004). At higher ploidy levels (8x, 10x), wild kiwiberry (*Actinidia* spp.) has high concentrations of certain compounds, like ascorbic acid and some amino acids (Zhang Y. et al., 2017). So far, only a few studies have investigated the occurrence and extent of metabolic alterations following polyploidization. Thus, the effect of ploidy on plant metabolism is still unclear.

## Polyploid Effects in Composite Plants

### Impact at the Rootstock Level and in Grafted Plants

**Figure 2:**
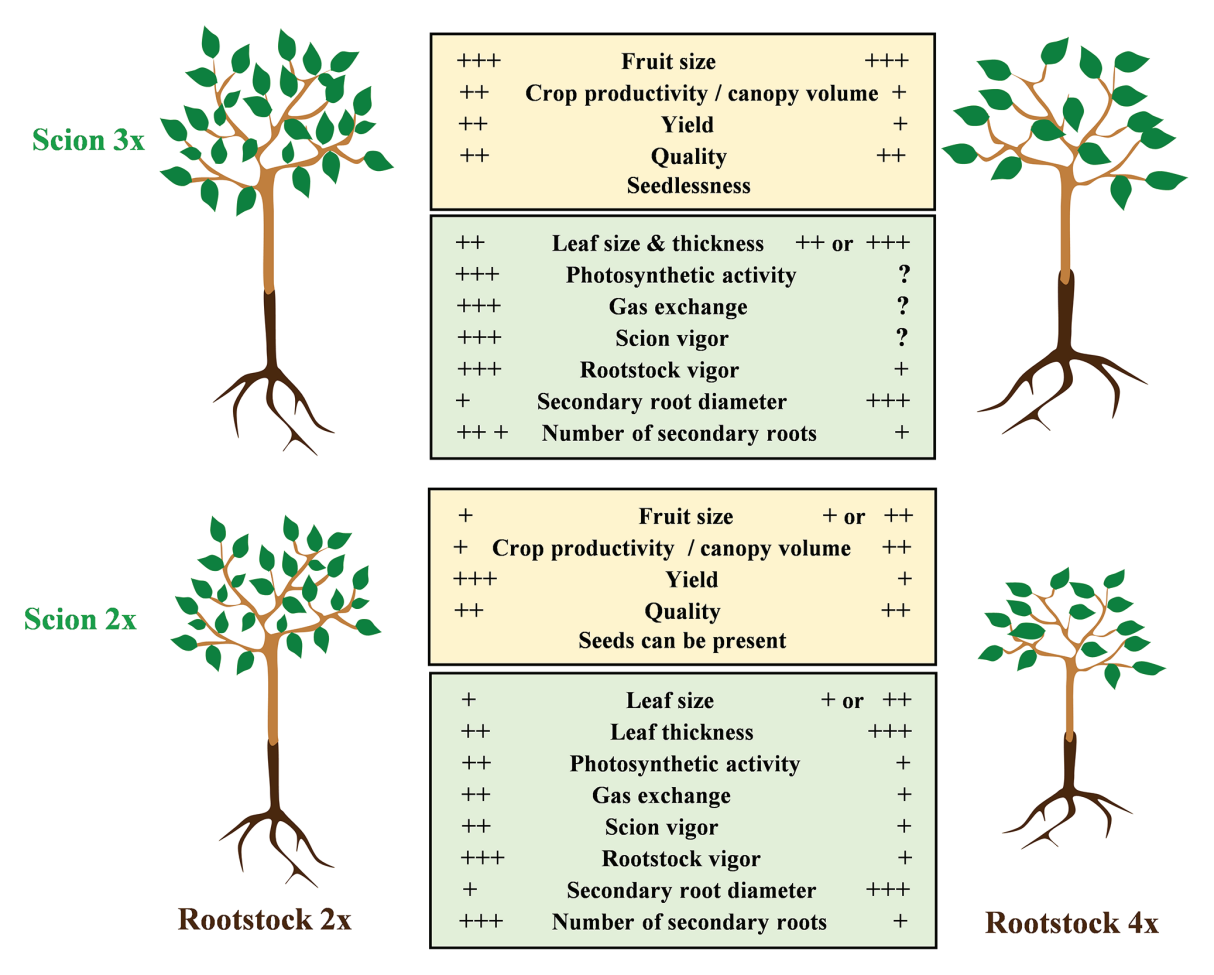
Physiological implication of the polyploidization when associating 2x and 3x scion with 2x or 4x rootstock. On one hand, 4x rootstock confers a more limited vigor than its respective 2x. On the other hand, a 3x scion will induce a greater vigor and larger leaves than in the 2x scion. At root level, tetraploidy will limit the tree vigor but will favor an increase of the size of the secondary roots. At the root and scion level, polyploidy may induce large phenotypical changes.

When used as rootstocks, polyploid plants can provide desirable attributes like vigor reduction or enhanced biotic and abiotic stress tolerance. Vigor reduction or dwarfism is one of the most sought-after phenotypes in rootstocks and is a very common effect of genome duplication on trees (Table 1). The decrease in tree volume, height, canopy diameter, and/or circumference reduces the need for pruning in commercial orchards and facilitates phytosanitary interventions. Interestingly, greater vigor was also found in sweet cherry (*P. avium* L.) grafted on 3x “Colt” rootstock (Webster et al., 1996). Tree vigor is known to be affected by numerous factors, including root hydraulic pressure, water uptake efficiency, hormone profile, nutrient uptake, and stomatal conductance (Warschefsky et al., 2016). The ploidy-driven changes in vascular anatomy may be the main factor underpinning the dwarfing effect. Increased cell size, reduced number of cells, changes in cell cycle duration, altered water transport capacity, or higher photosynthesis rates led by changes in leaf function and size have been suggested to trigger this differential growth pattern. These processes are regulated by phytohormones, whose levels have been proven to be altered by genome duplication (Dudits et al., 2016). Some 2x rootstocks can promote dwarfism in apple trees (Pilcher et al., 2008; Fazio et al., 2014). Polyploid rootstocks have been used commercially to reduce tree size, like citrus 4x somatic hybrid (allotetraploids) and 3x cherry rootstocks (4x *P. cerasus* L. cv. *Schattenmorelle* × 2x *P. canescens* Bois; Grosser and Chandler, 2000; Grosser et al., 2003; Prassinos et al., 2009). This can also be the case when using 4x rootstocks in citrus (Hussain et al., 2012) or tetraploid varieties in apple (Ma et al., 2016). This phenotype is most of the time associated with the translocation of some compounds such as hormones from the root to the grafted scion (Atkinson and Else, 2001), leading to gas exchanges regulation and anatomical changes (Allario et al., 2013) such as in leaf size and thickness or fruit size (Figure 2; see also Table 1). In 4x apple, decreased brassinosteroid and indole-acetic acid, which play a role in plant elongation, may also impact size reduction (Ma et al., 2016). In citrus, polyploid breeding over the last decades has successfully provided opportunities to select highly marketable dwarfing rootstocks (Grosser and Gmitter, 2011; Ollitrault and Navarro, 2012). Both autotetraploid and allotetraploid citrus often grow slower than their 2x counterparts and can induce dwarfism to the scion (Grosser et al., 2012; Ruiz et al., 2016a, 2018; Figure 2). In the end, the induced dwarfism allows high-density plantation and, in turn, facilitates orchard management (Figure 2). When used as rootstocks, 4x citrus do not reduce the scion’s yield efficiency nor do they have an impact on scion fruit quality parameters such as fruit size, yield, acids, or sugars. However, 4x rootstocks may have an impact on specific fruit compounds like phenolics or flavonoids (Hussain et al., 2012; Ruiz et al., 2016c). The use of 4x rootstock in association with clementine (*C*. × *clementina*) did not impact fruit quality (Hussain et al., 2012). Thus, citrus cultivated on 4x rootstocks produce fruits with excellent organoleptic qualities and can increase the profitability of plantations (Grosser et al., 2015). Sugars, organic acids, fatty acids, carotenoids, and flavonoids are the main drivers of fruit organoleptic quality (Ulrich and Olbricht, 2011), which is also highly influenced by the rootstock (Castle, 1995). Grafting allows us to take advantage of the rootstock’s better adaptation to abiotic and biotic stresses, which may improve plant development and yield production. For example, the 4x watermelon rootstock USVL-360 is an alternative to *Cucurbita* spp. rootstocks and provides vigor and resistance to the root knot nematode, while having similar yields to commercially available rootstocks (Levi et al., 2014).

From a breeding perspective, using polyploid genotypes on grafted crops to achieve even better tolerance to biotic stress is questioned. Breeding of grafted crops focuses on finding complementary traits for scions and rootstocks so they can be matched. Furthermore, an important part of the adaptation to variable abiotic and biotic environments is devoted to the rootstock.

**Figure 3:**
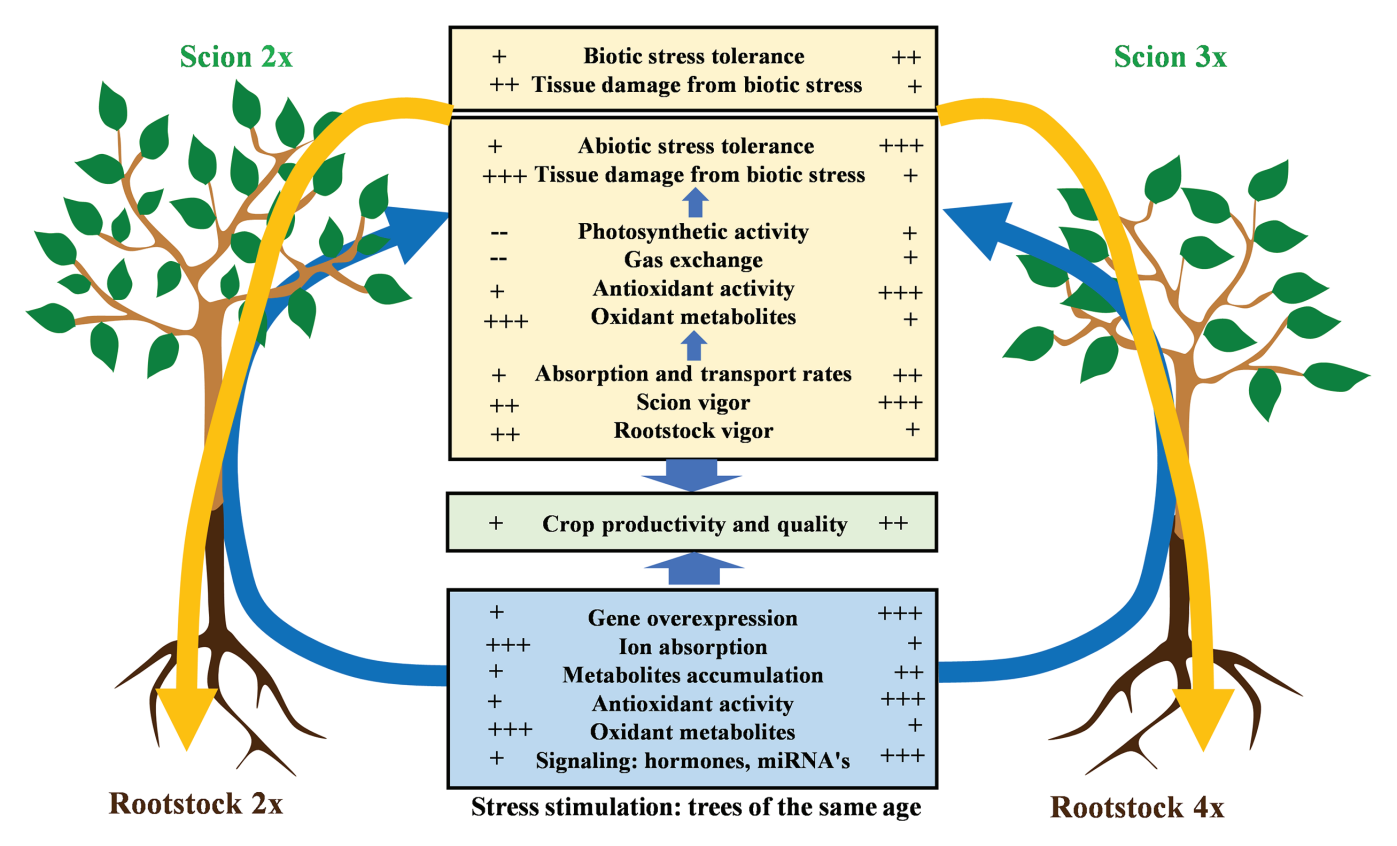
Physiological and molecular implications for stress tolerance when associating a 2x or 3x scion with a 2x or 4x rootstock. Arrows indicate the influence of the different factors at root and scion level depending on the ploidy that may change the physiology or the adaptation of the scion/rootstock association.

It is very difficult to find a rootstock that provides all the desired characteristics required by a crop production system. However, a good combination is important for the cultivation of a crop in a given available area. In some cases, the existence of a suitable rootstock is the decisive factor for cultivating in areas that are severely affected by environmental constraints (Figure 3), plant diseases, and pests (Reig et al., 2019). Moreover, the rootstock choice has long-term implications in tree crop cultivation, as it will strongly determine the profitability of the plantation. It is usually recommended that different suitable rootstocks are combined within each cultivation area to interfere with the spread of diseases and promote resilience (Koepke and Dhingra, 2013). Thus, generating variability and having a collection of rootstocks that is adequately phenotyped for disease resistance and stress tolerance is key for the long-term success of a crop cultivation in a given area.

### Grafting Compatibility and Impact of Polyploidy

Several reviews have been devoted to the grafting process and mechanisms (Mudge et al., 2009; Goldschmidt, 2014) and to the impact of the rootstock on the scion (Jensen et al., 2010; Koepke and Dhingra, 2013; Gautier et al., 2019). The grafting behavior of polyploid rootstocks or scions in association with classical 2x plant materials remains unexplored. In citrus, the use of 4x rootstocks with 2x or 3x scions that are compatible with the respective 2x rootstock does not seem to alter graft compatibility (Hussain et al., 2012; Allario et al., 2013). This can be explained by the genetic status of the 4x being identical to the 2x. However, due to the anatomical changes induced by polyploidy, such as increased cell size, an altered cell adhesion process could be expected. Interestingly, studies in tobacco (*Nicotiana* spp.) have shown that the entire nuclear genomes of both the scion and the rootstock can be transferred across the graft junctions to generate a novel 4x species (Fuentes et al., 2014). The plants regenerated by tissue culture from cells taken from the junction are true allotetraploid hybrids, underlying the possible interactions between different ploidy levels at the rootstock/scion intersection.

## Gene Expression Changes in Polyploid Crops and Functional Implications

Genome doubling driven by polyploidization modifies organization at the genetic and epigenetic levels. In comparison to artificial polyploids, natural polyploids of the same species have acquired novel characteristics thanks to polyploid lineage evolution (Semenuik and Arisumi, 1968; Lignowski and Scott, 1972; Lumaret, 1988; Levin, 2002). Meanwhile, the artificial ones may show superior performances relative to the natural ones, probably due in part to the strong selective pressure that the antimitotics, or other substances used to create the artificial autopolyploids, may impose. For example, the consequences of colchicine treatment may appear in the second generation of artificial polyploid plants (Münzbergová, 2017). Whether natural or artificially induced, genomic shock occurs mainly by the association of distant relatives by allopolyploidization and causes many genetic and/or epigenetic changes on gene expression. These include chromosome rearrangement, sub-functionalization and transposon activation, duplicate gene loss or gain, and gene activation or repression. Epigenetic changes induce variations at the gene expression level without altering the DNA sequence by modifying the DNA compaction or by interfering with RNA function (Comai, 2005; Madlung, 2013; Sattler et al., 2016). Nevertheless, the effect of genome fusion and gene duplication on gene expression at the transcriptome level differs according to whether the genome doubling comes from allopolyploidization or autopolyploidization (Renny-Byfield and Wendel, 2014). It has long been recognized that allopolyploids are more frequently observed in the wild than autopolyploids and that allopolyploids played a more important role in plant evolution. However, some studies have shown a higher incidence of autopolyploids than was previously thought (Soltis and Soltis, 1999; Soltis et al., 2007). The phenomenon of allopolyploidization alters transcription at the genome scale. Changes in gene expression are primarily affected by divergent genome hybridization and possible variation in genetic components rather than by a change in the ploidy level (Osborn et al., 2003; Auger et al., 2005; Wang et al., 2006). Conversely, in autopolyploids, the effect of genome doubling on gene expression is quite limited and does not show a linear relationship between the ploidy level and the transcriptional output. For example, citrus fruit of interspecific somatic allotetraploid showed around 4% transcriptome divergence between its two progenitors (Bassene et al., 2010). The difference reached up to 25% in allotetraploid cotton plants (Adams et al., 2003). Meanwhile, in mature leaves of “Yuzu” lime (*C. junos* Siebold ex Tanaka), “Rangpur” lime (*C*. × *limonia* Osb.), and mulberry (*Morus alba* L.), the differences observed in gene expression between 2x and autotetraploid genotypes are only 0.8, 1.08 and 2.87%, respectively (Allario et al., 2011; Dai et al., 2015; Tan et al., 2015).

Whether the genome doubling comes from allopolyploidization or autopolyploidization, the changes in gene expression affect a variety of biological processes in response to internal and external signals, such as, growth, development, and tolerance/resistance to both abiotic and biotic stresses. In addition, this differential gene expression depends on the organ, development stage, and environmental conditions (Chen and Ni, 2006; Doyle et al., 2008; Zhao et al., 2009).

Modifications of growth, development, and adaptation to abiotic and biotic stress in polyploid plants have been associated with modifications in gene expression, upregulation or downregulation of biosynthesis, transport, reception of primary and secondary metabolites (hormones, carbohydrates, organic acids, amino acids, and proteins, etc.), and enzymes. Polyploidy also changes the amount of signal molecules like small noncoding RNAs (sRNAs). These molecules regulate the expression of transcription and signaling factors related to cellular growth, development, and adaptation by regulating gene expression (Dhawan and Lavania, 1996; Ni et al., 2009; Jackson and Chen, 2010; Kim and Chen, 2011; Ng et al., 2012; Zhou et al., 2015; Javadian et al., 2017; Van Hieu, 2019). All the above mentioned molecules and metabolites circulate between the rootstock and scion through the vascular system, and have an impact either directly as a metabolite or indirectly as a signal to modulate the composite plant’s function (Notaguchi et al., 2008, 2015; Martínez-Ballesta et al., 2010; Goldschmidt, 2014; Albacete et al., 2015).

The regulation of phytohormones, such as cytokinin, ethylene, gibberellin (GA), brassinosteroids (BRs), and auxins plays a central role in genetic networks involved in altering plant growth (Table 1; Boss and Thomas, 2002; Wang et al., 2013b; Ayano et al., 2014). In polyploid plants, the variation of gene expression in comparison to 2x has been related to differential phytohormone concentrations. For example, the artificial autotetraploid mulberry (*Morus alba* L.) showed that compared to its 2x about 30 of these differentially expressed genes were associated with biosynthesis and transduction of phytohormones, such as cytokinin, GA, ethylene, and auxin involved in phenotypic changes (Dai et al., 2015). The changes in expression of key genes that affect the regulation of phytohormone pathways may lead to plant dwarfism. For instance, in 3- and 5-year-old plants of the artificial autotetraploid apple (*M*. × *domestica*), the altered gene expression modified the content of indoleacetic acid (IAA) and BRs leading to a decrease in tree growth resulting in a dwarfing effect. In addition, the accumulation of miR390 after genome duplication results in upregulation of short-acting apple RNA3 (MdTAS3), which in turn downregulates the expression of MdARF3, an auxin response factor (Ma et al., 2016). The dwarfing effect is also observed in other artificial autotetraploid apple trees prior to flowering; however, growth is restored after the juvenile phase (Xue et al., 2017). Rootstocks play a key role in controlling scion growth and development by the modulation of hormone signaling pathways (Cookson et al., 2013; Gregory et al., 2013; Berdeja et al., 2015; Corso et al., 2016; Adams et al., 2018). Indeed, in grafted plants, cytokines are synthesized in the rootstock and then transported into the apical meristem through the phloem, while ethylene is synthesized in the rootstock xylem. Conversely, auxin is synthesized in the scion’s leaves and is then transmitted to the roots *via* phloem (Zhao, 2018). The use of polyploid rootstocks or scions may alter the concentration and balance of phytohormones (ABA, IAA, BRs, auxin, and cytokinin) and impact the tree’s phenotype. In “Rough” lemon (*C. jambhiri* Lush.) grafted with autotetraploid “Commune” clementine, Apetala 2 (AP2)/ethylene element binding protein (EREBP) genes that play a role in regulating various developmental processes are repressed (Riechmann and Meyerowitz, 1998; Ninoles et al., 2015). When 2x “Delta Valencia” sweet orange [*C*. × *sinensis* (L.) Osb.] is grafted into 4x “Rangpur” lime, the root-to-shoot signaling, which is mediated by the constitutive expression of many genes, shows a differential pattern when compared to plants grafted into the corresponding 2x rootstock. Overexpression of the CsNCED1 gene, which is involved in regulating abscisic acid (ABA) biosynthesis, increases ABA transfer from the roots to the aerial part and modifies gas exchange at the scion level (Allario et al., 2013). Studies based on autopolyploid Paulownia [*P. fortunei* (Seem) Hemsl.] transcriptome found that many miRNAs are also predicted to participate in ABA signaling (Zhao, 2018; Figure 3). However, it is interesting to note that the phytohormone contents fluctuate throughout development, independently of ploidy, but a different pattern may predominate. In autotriploid and autotetraploid watermelon fruit, IAA is higher than in 2x during developmental stages. Meanwhile, no differences in ABA expression have been observed on the 3x watermelons. On the other hand, GA, the main cytokine zeatin riboside (ZR) and BR contents are lower than IAA and ABA and are gradually reduced during fruit development independently of the ploidy. The low concentrations of ZR and BR are thought to be due to a small impact of ploidy on the expression of related genes (Dou et al., 2017).

Organic acids acting as intermediates in the tricarboxylic acid (TCA) cycle (citric acid, malic acid, fumaric acid, and succinic acid), a central pathway of energy metabolites and anabolic precursors for plant proliferation and survival, are also affected by modifying the gene dosage. The main difference between 2x and autotetraploid Col-0 *Arabidopsis* metabolite accumulation is found in the TCA cycle and γ-amino butyric acid (GABA) shunt. TCA and GABA concentrations respond to a differential expression of their related genes (Vergara et al., 2016). Studies performed on mature leaves of mandarin (*C. reticulata* L.) grafted on 2x and 4x trifoliate orange (*Poncirus trifoliata* L. Raf.) rootstock, show an increase in few TCA compounds including citric acid, but a decrease in other secondary metabolites such as phenylpropanoids and terpenoids. The accumulation of primary metabolites appears to be related to epigenetic modifications rather than genetic variation. Thus, Tan et al. (2017) hypothesized that the decrease in secondary metabolites could indicate that primary metabolism takes priority to relieve the genomic stress encountered in the early stages of genome doubling, probably to allow better vitality and growth. Overall, the impact of polyploidization on metabolites differs depending on the species and specially on their roles as scions, rootstocks, or non-grafted plants (Figure 3). This behavior indicates that metabolic changes induced by polyploidization is associated to roostock-scion interactions (Fasano et al., 2016) and further research is needed to decipher stable patterns.

## Polyploidy Improves Stress Tolerance

Polyploidy, whether auto or allo is associated with enhanced tolerance to a wide range of stresses, including drought, salinity, cold, heat, nutrient deprivation, or excess light both in wild and cultivated plant species (Doyle and Coate, 2019). According to the abundant research on synthetic autopolyploids, genome doubling can generate variation *per se* in the absence of hybridity. There are widespread recurring patterns associated with genome duplication that lead to enhanced stress tolerance (Doyle and Coate, 2019). Most of the mechanisms described involve substantial changes in morphology but subtle changes in gene expression (Allario et al., 2013). However, altered hormone signaling (del Pozo and Ramirez-Parra, 2014), differential metabolic responses or altered DNA methylation patterns (Yu et al., 2009; Aversano et al., 2013) have also been identified on autopolyploids. These effects can be highly variable between species or even among individuals within the same species. For instance, Denaeghel et al. (2018) reported increased cold tolerance in autotetraploid *Escallonia rubra* (Ruiz and Pav.) Pers. when compared to the 2x. In contrast, autotetraploid *E. rosea* Griseb. does not differ from 2x in its cold tolerance. In artificial allopolyploids, that combine increased ploidy and hybridization effects, differential gene (Zhao et al., 2017) and protein (Yan et al., 2017) expression has been described more often. In this case, research has shown that it is possible to efficiently combine the desired parental phenotypes on the progenies (Grosser and Gmitter, 2011), although genome instability and allelic losses have also been described (Pensabene-Bellavia et al., 2015; Ruiz et al., 2018). Overall, polyploid breeding is progressively carving out its place as a method to improve crops for abiotic stress tolerance, as it opens the possibility of adding functional novelty, while combining genomes that are associated with a well-known and highly valued agronomic behavior. The outcome minimizes the risk that undesired behavior causes economic loss when compared to traditional breeding methods. In the following section, we review the effect of genome duplication on abiotic stress tolerance. Specifically, we will focus on the biochemical, morphological, and physiological modifications underpinning the enhanced tolerance of polyploid crops to a wide range of environmental stresses that have been described lately.

### Alterations in ROS Metabolism

Most abiotic stresses have a common impact on plants: they induce the accumulation of reactive oxygen species (ROS), which are by-products of the physiological metabolism. According to the abundant available literature, altered ROS metabolism is a common effect of polyploidization that helps to improve stress tolerance. In 4x *Arabidopsis* subjected to drought stress, ROS homeostasis is altered when compared to 2x, resulting in more efficient adaptation (del Pozo and Ramirez-Parra, 2014). Similarly, several polyploid crops have enhanced stress tolerance responses that are correlated with oxidative metabolism alterations. The increase in ROS is an immediate consequence of salt stress in plants, as they are involved in transcriptional regulation and ion flux alteration to improve the plant’s general performance. High levels of salinity generate damaging ROS as a part of the programmed cell death response (Isayenkov and Maathuis, 2019) and are balanced by ROS scavengers. In this sense, faster ROS production response in earlier stress phases and a more efficient ROS scavenging capacity in later stages have been described in Black locust (*Robinia pseudoacacia* L.; Meng et al., 2016; Luo et al., 2017) and different citrus species (Podda et al., 2013; Khalid et al., 2020) as a way to cope with salt stress. This effect has been widely documented in 4x citrus for a variety of stresses. For instance, 4x *P. trifoliata* subjected to drought has a transcriptome enriched in genes coding for enzymes related to antioxidant process, higher peroxidase (POD) and superoxide dismutase (SOD) activity, lower level of ROS, and less tissue damage. This mechanism in combination with osmotic adjustment has been described to enhance tolerance to drought when compared to the 2x (Wei et al., 2019). Furthermore, allotetraploidization might have a stronger effect on stress protection, combining two different genetic pools and the genome duplication effect. The allotetraploid hybrid FlhorAG1 (*C. deliciosa* Tan. + *P. trifoliata*) when compared to its parents and respective autotetraploids, had lower photoinhibition (Fv/Fm) and less accumulation of the oxidative markers malondialdehyde (MDA) and H_2_O_2_. This was correlated with a greater increase in some antioxidant activities during cold stress (SOD, ascorbate peroxidase or APX and glutathione reductase or GR) and light stress (SOD, APX, and monodehydroascorbate reductase or MDHAR; Oustric et al., 2018). Later studies on nutrient deprivation showed that the allotetraploid FlhorAG1 and several autotetraploid citrus genotypes are more tolerant to nutrient deficiency than their 2x counterparts. This behavior is related to enhanced photosynthetic capacity and a more favorable balance in their oxidative metabolism (Oustric et al., 2019a). The stress-protective effect driven by polyploidy was found to be graft-transmissible by different studies on citrus varieties grafted on 4x rootstocks. For instance, “Commune” clementine is more tolerant to cold stress when grafted on 4x “Carrizo” citrange (*P. trifoliata* × *C. sinensis*) than in 2x, as photosynthetic machinery stays more active and thus ROS production and damage are limited (Oustric et al., 2017). Lower levels of MDA, less electrolyte leakage and higher specific activities of catalase (CAT), APX, and dehydroascorbate reductase or DHAR were detected on plants grafted on 4x rootstocks, suggesting that a more efficient antioxidant activity promoted by the rootstock plays a role in their enhanced cold tolerance. Another example is the behavior of “Kinnow” mandarin plants grafted on three different rootstocks and subjected to chromium toxicity. In this study, plants grafted on 4x rootstocks were more tolerant than plants grafted on 2x rootstocks (Balal et al., 2017). This was attributed to more efficient accumulation of the metal on the 4x roots, and in turn, decreased transfer to leaves that prevented damage by accumulation. A more active antioxidant system was also identified in 4x roots.

Changes in primary and secondary metabolite expression, such as upregulation of sugars, amino acids, organic acids, and fatty acids driven by genome duplication have been reported to underpin the ROS metabolism alterations (Tan et al., 2015). The reason behind may be that large cells are disproportionately more productive than small cells. This might be driven by surface-to-volume ratio effects or increase in organelle number and size as described by Doyle and Coate (2019). These authors have also highlighted the potential contribution of ploidy-driven changes at the nucleolus, mitochondria, chloroplast, and endoplasmic reticulum on enhanced tolerance to abiotic stress. However, there is still a lack of knowledge in this area.

### Enhanced Tolerance to Drought and Salinity

One of the main mechanisms involved in plant drought tolerance is the ability to deal with cavitation is one of the mechanisms favoring plant drought tolerance (Brodersen and McElrone, 2013). In polyploid species, xylem vessels are usually bigger in diameter than in 2x counterparts due to bigger cell size (Soltis et al., 2014), suggesting that polyploidy may increase sensitivity to drought due to an increased vulnerability to water flow instability under tension (Tyree and Ewers, 1991; Maherali et al., 2009). However, there are reports of decreased sensitivity to low soil moisture among polyploids (Maherali et al., 2009; Hao et al., 2013; Zhang W. W. J. et al., 2017). This paradox may be explained going deeper into the complexity of the occurrence and reversion of embolism in plants. Cavitation is not a straightforward process; it is influenced by several other traits that are not commonly evaluated. Pit anatomy (Zhang W. W. J. et al., 2017), vessel wall surface properties, and associated fibers or the whole xylem architecture may play a fundamental role in cavitation propension (Rockwell et al., 2014; Guet et al., 2015). To our knowledge, these traits have not yet been explored on polyploid crops.

Equally important for drought tolerance are the changes in the transpiration rate. Stomata in polyploids are typically bigger and their distribution is less dense than in 2x (Hennig et al., 2015; Hïas et al., 2017; Corneillie et al., 2019), which may force lower stomatal conductance than 2x counterparts (Singh and Sethi, 1995; Syvertsen et al., 2000; Zhang et al., 2007; Allario et al., 2011) and improve photosynthetic efficiency (Warner and Edwards, 1993). In 4x “Hanfu” apple the enhanced photosynthetic capacity has a positive impact on fruit yield and quality (Xue et al., 2017). However, this effect seems to be dependent on scion polyploidy, as 4x rootstocks do not enhance the photosynthetic capacity of 2x scions (Oustric et al., 2017). It has been showed that stomata closure can depend on leaf ABA, even though rootstock may contribute to the production of ABA (McAdam et al., 2016). Polyploidy can lower the gas exchange capacity as seen in 4x strawberries (*Fragaria moupinensis* Cardot; Gao et al., 2017). In this sense, the stomata closure regulatory mechanism, led by root-to-shoot signaling that is triggered by drought and mediated by the plant hormone ABA (Schachtman and Goodger, 2008) is said to be altered by polyploidy. Autotetraploid “Rangpur” lime has higher constitutive production of ABA than the 2x counterpart, associated with increased drought tolerance (Allario et al., 2013). Likewise, stomatal closure in 4x *Arabidopsis* is more responsive to drought and ABA than in 2x (del Pozo and Ramirez-Parra, 2014), but there is also evidence of increased gas exchange capacity in 4x *Arabidopsis* (Monda et al., 2016). These findings suggest that additional ploidy-driven modifications at the root level may have a strong influence on the gas exchange process such as the root’s hydraulic conductivity (see next section). Overall, these stomata-related findings suggest that polyploids might provide better functional adaptation to drought stress, either when used as rootstocks or scions.

Furthermore, root hydraulic conductivity (Lp; m·s^−1^·MPa^−1^), which determines the root’s system water uptake capacity and plays an important role in water use, might be modified by ploidy. The main traits contributing to decrease Lp, both in woody and herbaceous species, are cortex width and the presence of suberin barriers, which manage the radial flow of water and solutes to the stele (Rieger and Litvin, 1999). Polyploids may have thicker root cortex and suberin depositions, determined by their bigger cell size and more active metabolism (Doyle and Coate, 2019), as it is the case of citrus (Syvertsen et al., 2000; Ruiz et al., 2016a,c) or Willow (Dudits et al., 2016), which would result in lower Lp than in 2x roots (Ruiz et al., 2016c). Decreased water uptake conserves the resource in the soil for longer periods and prevents root leakiness or backflow under dry conditions, thus delaying plant mortality. As an example, suberin barrier reinforcement contributes to reducing the transpiration rate, while increasing water-use efficiency in *Arabidopsis* (Baxter et al., 2009); this is a well-known adaptation mechanism that helps many annual and woody species overcome water stress (Barrios-Masias et al., 2015). Shorter, thicker and less branched roots that develop earlier and thicker suberin barriers have been identified on different 4x citrus rootstocks (Syvertsen et al., 2000; Ruiz et al., 2016a,c) and have been associated with lower hydraulic conductivity (Huang and Eissenstat, 2000). These modifications operate in “Carrizo” citrange to maintain unaltered leaf hydric status under osmotic stress, allowing gas exchange parameters to be sustained and limiting water consumption, while the 2x is drastically affected (Ruiz et al., 2015, 2016b; Oliveira et al., 2017). Similarly, 4x *Acacia* (*Acacia senegal* L. Willd) grew faster than 2x only under drought stress (Diallo et al., 2016). However, these anatomical root modifications have yet to be described in other polyploid crops.

The mechanisms described above are operative in the early stages of drought stress when water resources are in short supply to plant organs. Meanwhile, alternative local mechanisms may act in later stages to prevent dehydration and tissue damage. Hydraulic capacitance involves the use of tissue water storage to buffer local desiccation, maintain function (Huang et al., 2017; Vogel et al., 2017), or reverse cavitation (Brodersen et al., 2018). In that sense, polyploid plants may have an advantage over the 2x at preserving tissue water content as shown by the autotetraploid *Arabidopsis* detached rosette leaves (del Pozo and Ramirez-Parra, 2014). Similarly, autotetraploid “Gala” apple has the ability to delay drought-induced misperformance by trading hydraulic safety for increased release of capacitively stored water from living tissues (De Baerdemaeker et al., 2018). However, it is unknown whether bigger polyploid cells can accommodate bigger vacuoles increasing their water storage capacity.

Plant responses to salinity are induced by two different components of saline solutions. Initial adjustments are attributed to the water-stress effects triggered by the osmotic component of soil water potential. Ion-specific toxicity occurs later when saline ions accumulate causing tissue damage and abscission. Both effects reduce the plant’s photosynthetic capacity, first by dramatically decreasing stomatal and root hydraulic conductance to limit water use and second, by reducing the leaf area. Consequently, the plant must reduce its growth rate (Munns and Tester, 2008). At the later stage, plant mortality appears when toxic damage, poor water status, and starvation overcome tolerance capacity. Several polyploid crops respond more efficiently to soil water depletion aided by the different mechanisms mentioned above. These would also be operative to deal with both toxic and osmotic components of salt stress. In citrus, higher salt tolerance has been described in different 4x rootstocks in association with low water use (Saleh et al., 2008; Ruiz et al., 2016b,c) or low water availability (Mouhaya et al., 2010). The efficient regulation of water use in response to osmotic stress greatly prevents ion intoxication by excessive accumulation when uptake is proportional to water use (Moya et al., 2003). Conversely, this tolerant response could require a trade-off between fruit production and ion avoidance.

Also, important when dealing with salinity is the more balanced potassium-sodium (K^+^/Na^+^) homeostasis shown by polyploid plants. Maintaining high K^+^ uptake and tissue concentration is essential for salt tolerance (Wu et al., 2018), as K^+^ competes for similar ion channels, transporters, and active sites as Na^+^, preventing its accumulation and resulting functional disruption (Nieves-Cordones et al., 2010; Isayenkov and Maathuis, 2019). For example, polyploid *Arabidopsis* has greater tolerance to salinity, associated with higher K^+^ uptake and lower Na^+^ accumulation in leaves. Surprisingly, this effect has been shown to rely on rootstock polyploidy rather than on shoot cytotype (Chao et al., 2013). Hence, it is a root-dependent phenotype that could be provided to grafted crops using polyploid rootstocks. Higher K^+^ retention when faced with salt stress has been also observed in hexaploid bread wheat (*T. aestivum* L.; Yang et al., 2014), in “Carrizo” citrange (Ruiz et al., 2016b) and in the allohexaploid sweet potato wild relative *Ipomoea trifida* (Kunth) G. Don (Liu et al., 2019). However, the reasons behind this ploidy-driven differential cation regulation are unknown.

Most crops suffer from the effects of salt toxicity, mainly associated with Na^+^ leaf accumulation (Munns and Tester, 2008). However, some tree crop species are mostly affected by leaf chloride (Cl^−^) accumulation instead, such as avocado (*Persea americana* Mill.; Acosta-Rangel et al., 2019), grape (Henderson et al., 2014), or citrus (Moya et al., 2003). Moreover, independent of which ion is the most damaging, the key for salt tolerance is the exclusion ability that some species have to limit ion uptake and translocation.

On the root apoplastic pathway, barriers to toxic ions are mainly based on suberin depositions at the exodermis and endodermis layers, whose presence/absence patterns are a determining factor for water and ion flow. In 4x citrus, enhanced Cl^−^ and boron exclusion ability has been related to a less branched and thicker root that develops earlier and thicker suberin barriers that are more restrictive to ion flux than in 2x counterparts (Ruiz et al., 2016a,c). Another essential mechanism used to deal with salinity is excess ion partition. Although this is not well understood, the three main compartments for Na^+^ and Cl^−^ allocation are under discussion. Most data suggest that plants prevent excessive Na^+^ and Cl^−^ accumulation in the cytosol and apoplast, to stop them from reaching concentrations beyond 50–80 mM to avoid hydric, biochemical, and nutritional imbalance (Isayenkov and Maathuis, 2019). In contrast, vacuoles can endure 10-fold higher ion concentrations than the later compartments. In this sense, polyploids would theoretically be at an advantage because of the ability to allocate, exchange, and dilute ions in their bigger vacuoles (Doyle and Coate, 2019). However, whether this mechanism plays a role in enhancing salinity tolerance has not been described on polyploid plants yet.

### Does Polyploidy Induce Better Tolerance to Biotic Stress?

In earlier sections of this review, some polyploids were shown to be more tolerant to abiotic stress induced by genome expression regulation leading to changes in physiological traits, hormonal production, or better antioxidant systems. QTL analysis revealed quantitative resistance for *Phytophthora infestans* and *Tecia solanivora* in 4x potato (Santa et al., 2018). Tetraploid and 3x banana cultivars had little damage due to nematodes and did not significantly reduce their plant height (Dochez et al., 2009). All the cultivated citrus are sensitive to Huanglongbing (HLB), a disease caused by the bacterium Candidatus *Liberibacter* sp. In citrus, HLB leads to an increase of callose synthesis at the sieve plate of the phloem cells, which will cause plugging of the pores (Koh et al., 2012), and thus will stop the symplastic transport between phloem cells. In the short term, the tree’s physiology will be greatly affected which will rapidly lead to the tree’s death. “Persian” lime (*Citrus* × *latifolia* Tan. ex Jimenez), which is 3x, is the most tolerant variety to HLB. It is thus possible that the better tolerance in “Persian” lime is related to the larger phloem vessels induced by polyploidy, which in turn will contribute to maintaining the flux of the phloem sieve for a longer time.

## Concluding Remarks

We believe that the research into synthetic polyploids in agriculture is still too limited. Overall, the mechanisms that contribute to stress tolerance may also play a role in keeping plant productivity and fruit quality during environmental constraints or intentional water shortage. For this reason, polyploid scions and rootstocks might be a convenient choice for promoting efficient and sustainable agriculture. As an example, grape, olive, and citrus are very relevant for evaluating the impact of polyploidy on plant stress adaptation since they are clonally propagated crops (De Ollas et al., 2019). Extensive studies are still required to decipher the impact of polyploidy at the rootstock or scion level on agronomical phenotypic traits. Additionally, better understanding of the associated genome expression regulatory mechanisms induced by polyploidy is required as well. Overall, the production of 3x and 4x genotypes is quite straightforward nowadays and numerous grafted plants of agronomic interest could benefit from the ploidy-induced novel phenotypes.

## Author Contributions

MR, JO, and RM outlined and prepared the first draft of the manuscript. MR, JO, JS, and RM wrote the manuscript and produced the tables and figures. All authors revised the subsequent drafts of the manuscript and approved the final version.

### Conflict of Interest

The authors declare that the research was conducted in the absence of any commercial or financial relationships that could be construed as a potential conflict of interest.

## Abbreviations

NAD-IDH: Isocitrate dehydrogenase
GABA-T: ɣ-aminobutyric acid transaminase
GABP: ɣ-aminobutyric acid permease
ABA: Abscisic acid
ACO2: Assimilation rate
APX: Ascorbate peroxidase
BRs: Brassinosteroids
CAT: Catalase
DHAR: Dehydroascorbate reductase
E: Transpiration rate
GA: Giberellic acid
GPX: Glutathione peroxidase
GR: Glutathione reductase
GS: Stomatal conductance
GSH: Glutathione
GST: Glutathione S-transferase
IAA: Indole acetic acid
Lp: Root hydraulic conductivity
MDHAR: Monodehydroascorbate reductase
POD: Peroxidase
PRX: Peroxiredoxin
QTL: Quantitative trait loci
ROS: Reactive oxygen species
SOD: Superoxide dismutase
